# Social Connection and Brain Function: From Measurement to Clinical Trials

**DOI:** 10.1093/geroni/igaf122.903

**Published:** 2025-12-31

**Authors:** Hiroko Dodge, Brea Perry

## Abstract

Over five decades of gerontological studies have shown the negative impact of social isolation on health, including an increased risk of dementia. The most recent Lancet Commission on Dementia reported that social isolation contributes to 5% of dementia cases, higher than the 2% attributed to physical inactivity or diabetes. Reducing social isolation could significantly impact dementia prevalence. However, compared to other behavioral interventions like exercise and diet, fewer randomized controlled trials (RCTs) have been conducted on social isolation/connectedness. Social isolation is multifactorial, affecting cognition through various pathways, including tangible and intangible factors, multiple types of cognitive stimulation, and emotional bonding. Thus, RCTs require careful consideration of theoretical frameworks to clarify and identify the different potential mechanisms. This symposium aims to highlight key factors in developing behavioral interventions for social isolation/connectedness through four presentations: 1) Perry will discuss egocentric social network methodology to assess social connectedness, which operationalizes social bridging and bonding theories. 2) Yu will present the results of her systematic literature review on psychosocial and physiological pathways linking social isolation/connectedness to brain health. 3) Krendl will examine whether the theory of mind mediates the relationship between older adults’ social connectedness and cognition, both cross-sectionally and longitudinally. 4) Dodge will present the theoretical framework and findings from the I-CONECT project, which aimed to enhance cognition by providing frequent conversational interactions as cognitive stimulation among socially isolated older adults. Together, this symposium will offer critical components for developing targeted interventions that address social isolation/connectedness and support cognitive resilience.

